# Construction and validation of a novel diagnostic model for esophageal squamous cell carcinoma: an integrated analysis of multi-omics data

**DOI:** 10.3389/fimmu.2026.1685902

**Published:** 2026-02-13

**Authors:** Yiyuan Cui, Sicong Li, Zhibin Wu, Yue Jin, Jingjie Yu, Yufan Chen, Jiayang Chen, Jinyuan Chang, Yijing Yan, Xinyu Li, Nuo Li, Shengjuan Hu, Chenxin Zhu, Li Feng

**Affiliations:** 1 Department of Traditional Chinese Medicine (TCM), National Cancer Center/National Clinical Research Center for Cancer/Cancer Hospital, Chinese Academy of Medical Sciences and Peking Union Medical College, Beijing, China; 2 Department of Oncology and Hematology, Dongzhimen Hospital/First School of Clinical Medicine, Beijing University of Chinese Medicine (BUCM), Beijing, China

**Keywords:** diagnostic model, esophageal squamous cell carcinoma, logistic regression, single-cell RNA analysis, weighted gene co-expression network analysis

## Abstract

**Objective:**

Esophageal squamous cell carcinoma (ESCC), highly prevalent in China, has a limited number of ideal genes for early diagnosis, highlighting the need for the development of novel biomarkers to improve detection capabilities. The purpose of this study is to develop and validate a new genetic diagnostic model for ESCC.

**Materials and methods:**

Publicly available bulk RNA-seq datasets (GSE23400, GSE17351, GSE20347) were merged to identify differentially expressed genes (DEGs) between ESCC and adjacent normal tissues. Weighted gene co-expression network analysis (WGCNA) and protein-protein interaction (PPI) were performed to identify hub genes associated with ESCC. We identified the intersecting genes between the DEGs and those within the ESCC-related module identified by WGCNA. We subsequently refined these intersecting genes via LASSO regression and then constructed a diagnostic model for ESCC using multivariate logistic regression. ESCC samples from the TCGA database were used as the external validation set. Validation of the identified protective factor was conducted through Western blotting (WB) in mouse ESCC models and immunofluorescence (IF) in human tissues. Additionally, single-cell RNA analysis was conducted to explore the cell types expressing the marker genes.

**Results:**

113 upregulated and 173 downregulated genes were found in the ESCC groups. WGCNA identified the blue module (13 genes) as most correlated with ESCC. We obtained a total of 13 intersecting genes. Among them, five genes formed the diagnostic model: Logit(P) = −24.4547 + 2.0567×*BID* + 0.7396×*CBX3* + 2.3757×*ECT2* + 0.5667×*KIF14* − 2.1019×*SORBS2*. The model achieved AUCs of 0.99 (training set) and 0.97 (external validation set). *SORBS2* was the only potential protective factor in the model. WB indicated higher expression levels of SORBS2 in the adjacent normal esophageal tissue compared to those in the ESCC tissue. single-cell RNA analysis revealed that myofibroblasts are the predominant cellular source of *SORBS2* expression within ESCC tumor tissue. IF confirmed lower level of SORBS2 expression in myofibroblast in the ESCC than those in the adjacent normal esophageal tissue.

**Conclusion:**

We developed an ESCC diagnostic model and identified *BID*, *CBX3*, *ECT2*, *KIF14*, and *SORBS2* as robust ESCC biomarkers. *SORBS2* is a tumor-suppressor gene predominantly expressed in myofibroblasts.

## 1 Introduction

Esophageal cancer is a malignant tumor that poses a serious threat to human health, ranking seventh globally in incidence and sixth in mortality (1). According to global cancer statistics for 2020, China accounted for 53.70% of new esophageal cancer cases and 55.35% of deaths worldwide (2). Notably, esophageal squamous cell carcinoma (ESCC) represents over 95% of cases in China, in stark contrast to Western countries where esophageal adenocarcinoma (EAC) predominates. Unfortunately, ESCC patients often exhibit no obvious symptoms in the early stages, and most are diagnosed only after experiencing dysphagia or metastatic symptoms (3). The majority of diagnosed ESCC patients are in the middle or late stages of the disease, with an overall five-year survival rate of only 6%–15% (4). If detected early and treated promptly, patients’ survival rates would significantly improve (2).

At present, reliable early-stage tumor markers and diagnostic modalities for ESCC remain markedly insufficient (5). Conventional tumor markers—such as squamous cell carcinoma antigen, carcinoembryonic antigen, and cytokeratin 19 fragment—are insufficiently specific or sensitive to serve as standalone diagnostic biomarkers for ESCC (6, 7). MicroRNAs show potential for early ESCC detection, but existing diagnostic models remain limited. For instance, miR-146a exhibits a specificity of only 68.6% and a sensitivity of 85.7% in discriminating ESCC (8), whereas serum miR-1246 offers 71.3% sensitivity and 73.9% specificity (9). Therefore, it is particularly important to develop new diagnostic methods to achieve early detection of ESCC.

Most tumor diagnostic models are constructed primarily with data from the TCGA database, which is overwhelmingly composed of samples from European descent (10). However, the high-risk population for ESCC predominantly resides in Asia, particularly China and Japan (11). Only relying on TCGA data to develop an ESCC diagnostic model introduces notable limitations. The GSE23400, GSE17351, and GSE20347 datasets predominantly comprise samples from Chinese and Japanese populations (12–14). Constructing ESCC diagnostic models using these datasets and identifying tumor suppressor genes could offer valuable insights for ESCC prevention and therapeutic strategies.

In this study, we screened five genes significantly associated with ESCC based on the GEO database, constructed a diagnostic model and nomogram, supported by experimental evidence showing differential expression patterns of these genes between tumor and normal tissues in animal models, complemented by human ESCC tissue immunofluorescence analysis of protective factors. In addition, by integrating single-cell RNA analysis tools, we delved deeply into the biological processes that genes might be involved in, providing potential insights for the clinical treatment of ESCC patients.

## 2 Materials and Methods

### 2.1 Study design

**Figure 1:**
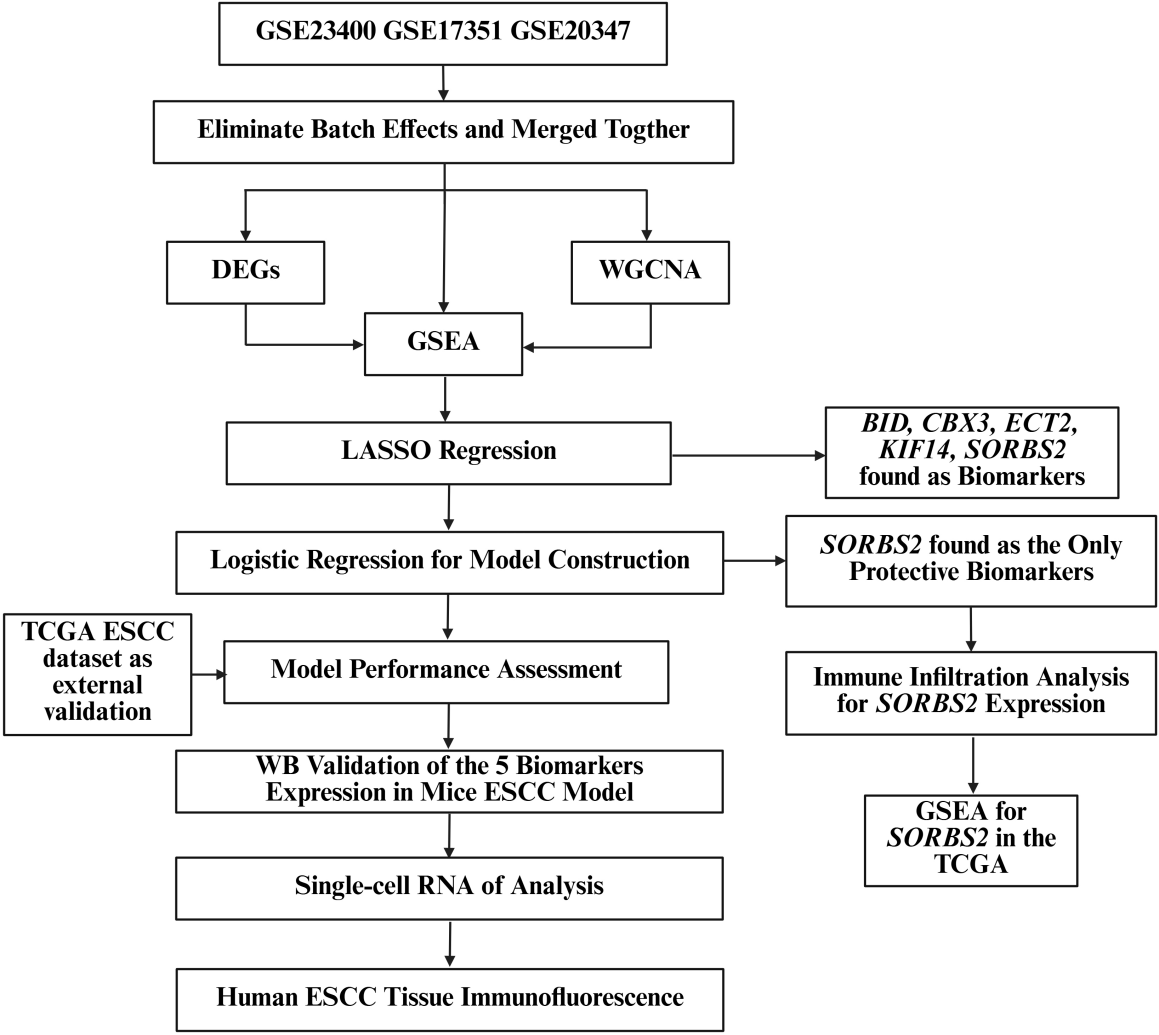
The workflow of the study. WGCNA, weighted gene co-expression network analysis; DEGs, differentially expressed genes; GSEA, Gene Set Enrichment Analysis; GO, Gene Ontology; KEGG, Kyoto Encyclopedia of Genes and Genomes; ESCC, esophageal squamous cell carcinoma; LASSO, the least absolute shrinkage and selection operator; *BID*, BH3-interacting domain death agonist; *CBX3*, chromobox protein homolog 3; *ECT2*, epithelial cell transforming sequence 2; *KIF14*, kinesin family member 14; *SORBS2*, sorbin and SH3 domain-containing protein 2; TCGA, The Cancer Genome Atlas.

The workflow of our study was shown in **Figure 1**. To obtain the ESCC sample, we retrieved and integrated three GEO datasets (GSE23400, GSE17351, and GSE20347) after batch effect removal. We performed DEG to identify differentially expressed genes between ESCC and adjacent normal tissues. WGCNA was employed to delineate gene modules most strongly associated with ESCC, and the intersection of WGCNA and DEGs refined the ESCC-related gene set. To identify the key pathways and biological processes, we conducted the GSEA for the merged dataset, DEGs and WGCNA. Potential biomarkers were identified using LASSO regression, followed by the construction of an ESCC diagnostic model via logistic regression and external validation in the TCGA ESCC dataset. To characterize the immune microenvironment of the tumor versus adjacent normal tissues, the immune infiltration analysis was conducted. The influence of the protective gene SORBS2 on the immune microenvironment was specifically examined, and GSEA further explored its functional roles. To validate the differential expression of candidate biomarkers between normal and tumor tissues, we established a Mice ESCC Model and carried out the WB experiment. Single-cell RNA analysis was performed to explore the cell type of tumor tissues and the potential functions of the protective gene. Finally, immunofluorescence staining of human ESCC specimens was carried out to confirm SORBS2 protein expression and localization.

### 2.2 Data acquisition and pre-processing

#### 2.2.1 Bulk transcriptome datasets

All datasets in this study were downloaded from the GEO database (https://www.ncbi.nlm.nih.gov/geo/). The GSE23400 dataset includes a total of 53 adjacent normal esophageal tissues and 53 ESCC tissues. The GSE17351 dataset comprises a total of 5 normal and 5 ESCC tissues. In the GSE20347 dataset, there are a total of 17 adjacent normal tissues and 17 ESCC tissues.

#### 2.2.2 Elimination of batch effects and data merging

The Robust Multiarray Average (RMA) technique was applied to correct background noise, normalize signals, and calculate expression values (15, 16). The inSilicoMerging package (v1.34.0) within the R (v4.3.1) software environment was utilized to integrate the three datasets above (17). Following this, the strategy was implemented to remove batch effects, leading to a consistent expression matrix for both normal and esophageal tissues (18).

### 2.3 Identification of ESCC-relevant genes

#### 2.3.1 Differentially expressed genes

We evaluated the distribution of gene expression levels in different samples in GSE23400, GSE17351 and GSE20347. Principal component analysis (PCA) and sample hierarchical clustering tree were applied to explore the intergroup difference and intragroup sample duplications. Abnormal samples were removed from subsequent analysis. The limma package (v3.56.0) was used for screening the DEGs with the cutoff criteria of *P* < 0.05 and |log2FC|>0.585, which was further visualized by pheatmap (v1.0.12) and ggplot2 (v3.5.1) packages in the heatmap and volcano map.

#### 2.3.2 Gene ontology and kyoto encyclopedia of genes and genomes enrichment analysis on DEGs

Gene Ontology (GO) analysis was performed utilizing the clusterProfiler R package (v4.9.0). For the functional analysis, parameters were configured with a significance level of *P* < 0.05, leading to the identification of molecular functions (MFs), biological processes (BPs), and cellular components (CCs). Furthermore, the clusterProfiler package (v4.9.0) was employed to set a *P* < 0.05 for functional analysis. This methodology enabled the identification of enriched signaling pathways associated with genes that may serve as potential therapeutic targets, as revealed through KEGG functional enrichment analysis (19).

#### 2.3.3 Weighted gene co-expression analysis

The WGCNA R package (v1.72-2) was utilized to create a co-expression network encompassing all genes from both ESCC and normal tissue samples. For further analysis, genes with variances of up to 50% were selected. The co-expression matrix was generated by determining the Pearson correlation coefficient between genes. Subsequently, an adjacency matrix was formulated using the equation *a_mn_* = |*c_mn_*|^β^, where *a_mn_* denotes the adjacency between genes *m* and *n*, *c_mn_* is the Pearson correlation coefficient, and β represents the soft threshold power. This weighted adjacency matrix was then converted into a topological overlap measure matrix to assess the genes’ network connectivity. The clustering dendrogram of the matrix was generated through the application of average linkage hierarchical clustering. A minimum gene module size of 30 was set to identify the appropriate module, and a threshold of 0.25 was established for merging similar modules.

#### 2.3.4 Gene set enrichment analysis on protective gene against the ESCC

In this research, GSEA was executed using the Molecular Signatures Database (MSigDB) Collection (c2.all.v7.0.entrez.gmt) via the clusterProfiler package (v4.9.0). The aim was to identify significant differences in pathways between ESCC or normal tissues. The distinction between ESCC or normal tissues served as the phenotypic label for the analysis, with the number of permutations configured to 1000. The remaining parameters were left at their default settings. Pathways with an adjusted *P* < 0.05 and an FDR < 0.25 were deemed to be significantly enriched.

#### 2.3.5 Immune cell infiltration analysis for genes protective against ESCC

CIBERSORT is a computational tool capable of inferring gene expression profiles and calculating the relative abundance of various cell types within a heterogeneous cell population based on gene expression data (https://cibersortx.stanford.edu). We utilized CIBERSORT to assess the relative proportions of 22 distinct immune cell types. Furthermore, we applied the two-sample *t*-test to explore differences in immune cell infiltration between the normal and ESCC groups, and between the low-expression SORBS2 group and the high-expression SORBS2 group.

### 2.4 Construction and performance assessment of a novel diagnostic model

#### 2.4.1 Sample size calculation

To achieve a power of 80% with a two-sided α of 0.05 and an assumed odds ratio (OR) of 2.1, we calculated the required sample size using the powerMediation package (v0.3.4) in R (v4.5.0). Assuming a 1:1 ratio between the ESCC group and the normal control group, the total sample size required was calculated to be 245 individuals.

#### 2.4.2 Construction and performance assessment of a novel diagnostic model

We developed a new diagnostic model using gene expression data from 126 ESCC tissue samples and their adjacent normal tissues in the merged GEO cohort, comprising a total of 252 samples across both groups. Initially, we employed the LASSO technique from the glmnet package (v4.1-9) to select genes closely associated with ESCC to prevent model overfitting, with 50-fold cross-validation. Subsequently, a diagnostic model was constructed using multivariate logistic regression based on the biomarkers identified by LASSO, and it was graphically represented as a nomogram using the rms package (v6.3.0). To quantify over-fitting, we performed 1,000 bootstrap resamples within the training set (n = 173) and calculated optimism-corrected R². Additionally, we extracted all of the ESCC samples, a total of 91 samples, from the TCGA database to serve as an external validation set. Ethnicity information for TCGA-ESCC samples was extracted from clinical metadata, with self-reported race categorized as asian, white, black or african American, or unknown. We assessed the model’s predictive performance on the training and external validation sets through ROC curve analysis, with evaluation metrics including AUC value, precision, specificity, sensitivity, positive predictive value (PPV), and negative predictive value (NPV). We also used calibration curve analysis to assess the consistency between predicted probabilities and the actual frequency of ESCC occurrence, and employed decision curve analysis (DCA) to evaluate the clinical utility of the nomogram.

### 2.5 Validation of the differential expression of marker genes in mouse ESCC tissues

#### 2.5.1 ESCC cell culture

The mouse ESCC cell line AKR were purchased from Cellverse Co., Ltd. Cells were cultured in DMEM supplemented with 10% fetal bovine serum and 1% penicillin–streptomycin until a confluent monolayer formed. When cells were grown at a density suitable for passage, they were trypsinized and passaged at a 1:3 ratio, and incubated under conditions of 37 °C and 5% CO_2_. After centrifugation, 4 × 10^6 AKR cells were resuspended in a mixture of PBS and Matrigel (4:1 ratio), where PBS was purchased from Beyotime Biotechnology, China, and Matrigel from Corning, USA.

#### 2.5.2 Construction of an ESCC mouse model

All animal experiments conducted in this study received approval from the Animal Experiment Ethics Committee of Cancer Hospital, Chinese Academy of Medical Sciences (Approval No. NCC2024A559). Eight 5-week-old male C57BL/6 mice (Beijing HFK Bioscience Co., Ltd) were acclimated for 7 days and then randomly divided into two groups of four: the ESCC group and the normal group. In the ESCC group, 1×10^6 AKR cells were injected subcutaneously into the right axillary region of each mouse to establish a mouse model of ESCC. Tumor size was measured from the third day. Tumor volume was calculated using the formula: V = 1/2 × (length × width^2) and tumor weight was recorded during necropsy. On the 10th day post-inoculation, mice were euthanized in a CO_2_ chamber. Tumor tissues were immediately collected from ESCC mice, and normal esophageal tissue was harvested from controls.

### 2.6 Western blotting validation of the 5 biomarkers expression

Flash-frozen ESCC tumors or adjacent normal epithelium (20 mg, n = 4 per group) were minced on ice and lysed in 150 µL ice-cold RIPA buffer (CST #9806) containing protease and phosphatase inhibitors plus 3 mm steel beads. Tissue was homogenized at 60 Hz for 5 cycles and centrifuged at 12–000 g, 4 °C for 20 min. Supernatants were quantified with a BCA kit (Sigma-Aldrich, V900933) and adjusted to 2 µg/µL with lysis buffer. Equal protein (20 µg) was mixed with 5 × Laemmli buffer, denatured at 100 °C for 15 min, loaded onto 10% SDS-PAGE gels (with 5% stacking gel), and electrophoresed at 120 V until bromophenol blue reached the gel bottom. Proteins were transferred to 0.22 µm PVDF membranes via wet transfer, blocked with 5% skim milk in TBST for 1 h at room temperature (RT), and incubated overnight at 4 °C with primary antibodies: anti-*BID* (1:1000, ab317809, Abcam), anti-*CBX3* (1:1000, ab213167, Abcam), anti-*ECT2* (1:2000, ab236502, Abcam), anti-*KIF14* (1:1000, PA5-68145, ThermoFisher), anti-*SORBS2* (1:500, PA5-67869, ThermoFisher). After three 5-min TBST washes, membranes were incubated with HRP-conjugated goat anti-rabbit IgG (1:5000, Zhengneng Biotech, 511103) for 1 h at RT. Signals were developed with ECL (Millipore, WBKLS0500) and imaged using a Liuyi Instruments chemiluminescence imager (WD-9423C). Band densities were quantified in ImageJ, normalized to β-actin, and plotted as mean ± SEM using GraphPad Prism.

### 2.7 Single-cell RNA sequencing data acquisition and analysis

Public scRNA - seq datasets (GSE196756) were retrieved from GEO and processed using Seurat (v4.0.4). Raw reads were aligned to GRCh38 using Cell Ranger. Cells under the following conditions were not included in this study (1): Cells outside the 1,000–35,000 detected-gene counts were discarded (2); Cells with <200 or >7,500 detected RNA counts were removed (3); Any cell whose mitochondrial transcripts exceeded 10% of its total gene counts was filtered out. Batch effects were corrected using Harmony. Dimensionality reduction was performed via PCA and UMAP. Clustering used Louvain algorithm (resolution = 0.2). Cell types were annotated by canonical markers. Differential expression analysis (Wilcoxon test, p < 0.05, |log2FC|>0.25) identified DEGs between clusters. Cell - cell communication was predicted by cellchat.

### 2.8 Immunofluorescence assay

The acquisition of all human tissues in this study was approved by the Ethics Committee of Cancer Hospital, Chinese Academy of Medical Sciences (Approval No. 23/510-4253). Formalin-fixed paraffin-embedded (FFPE) sections (5 µm) were dewaxed in xylene (3 × 5 min), rehydrated through graded ethanol (100%→95%→80%→70%; 3 min each), and microwave-heated in 10 mM citrate buffer (pH 6.0, 95 °C, 15 min). After cooling to RT, sections were blocked with 5% bovine serum albumin (BSA/PBS) for 30 min at 25 °C. Co-incubation with primary antibodies—transgelin/SM22 Polyclonal antibody (1:200, 10493-1-AP, proteintech) and *SORBS2* Polyclonal antibody (1:500, 24643-1-AP, proteintech)—proceeded overnight at 4 °C in a humidified chamber. Following triple PBS washes (5 min each), sections were incubated with Alexa Fluor 488-conjugated goat anti-mouse IgG (1:500, A-11001, Invitrogen) and Alexa Fluor 594-conjugated goat anti-rabbit IgG (1:500, A-11012, Invitrogen) for 1.5 h at RT in the dark. After extensive PBS washing, slides were mounted with DAPI-containing anti-fade medium (ProLong™ Gold, P36930, Thermo Fisher), cured overnight at 4 °C in darkness, and imaged using a Zeiss LSM 900 confocal microscope with Plan-Apochromat 63×/1.40 Oil objective. Sequential scanning (405/488/594 nm lasers) prevented spectral crosstalk. Quantitative analysis of fluorescence intensity was performed on ≥5 randomly selected fields per sample using ImageJ (v1.53k, NIH) with region-of-interest (ROI) measurements after background subtraction.

### 2.9 Statistical analysis

All bioinformatics analyses were performed in R v4.3.1. Key packages included: limma v3.56.0 for differential expression analysis; WGCNA v1.72–2 for co-expression network construction; ggplot2 v3.5.1 for data visualization; inSilicoMerging v1.34.0 for batch effect correction. Experimental data were presented as mean ± standard deviation (SD). For comparing data between two groups, a *t*-test was applied, while for comparisons among multiple groups, one-way analysis of variance (ANOVA) was used. Statistical analysis and graphing were conducted using GraphPad Prism 10 software. A P-value < 0.05 indicates a statistically significant difference.

## 3 Results

### 3.1 DEGs revealed 113 significantly up-regulated and 173 significantly down-regulated genes

**Figure 2:**
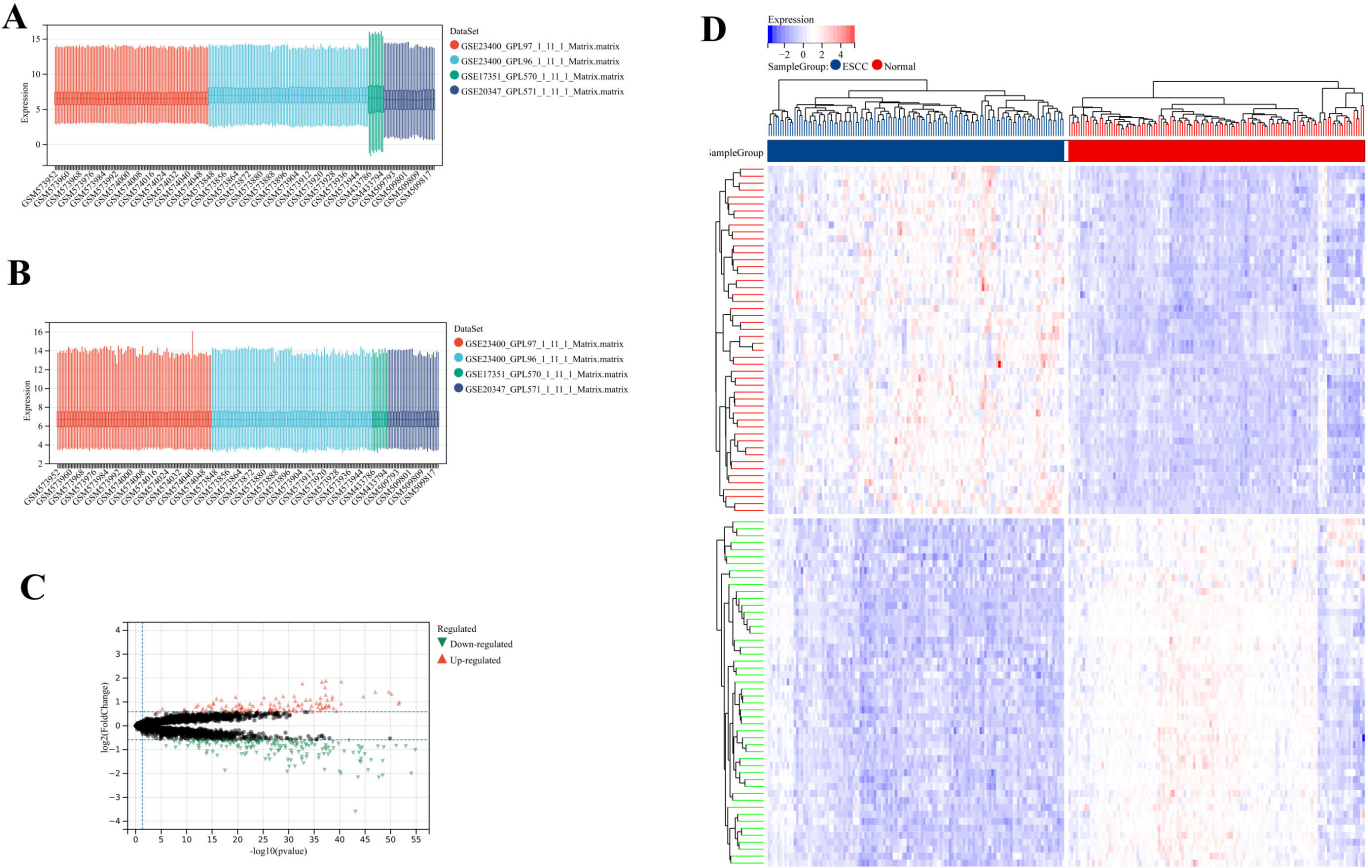
Integrative analysis of DEGs. **(A, B)** The box plots of the merged database. **(C)** Volcano map of DEGs. **(D)** Heatmap of DEGs.

As shown in **Figures 2A, B**, the sample distributions across datasets exhibited notable differences before batch effect removal, as evidenced by box plots, which underscored the presence of batch effects. Post-removal, the data distributions across datasets became more consistent. Through fold change (FC) and P-value filtering (|log2FC|>0.585 and P<0.05), a total of 113 genes were found to be significantly upregulated and 173 genes significantly downregulated in squamous carcinoma tissues (**Figures 2C, D**).

### 3.2 GSEA for the DEGs and merged dataset revealed key pathways and biological processes

**Figure 3:**
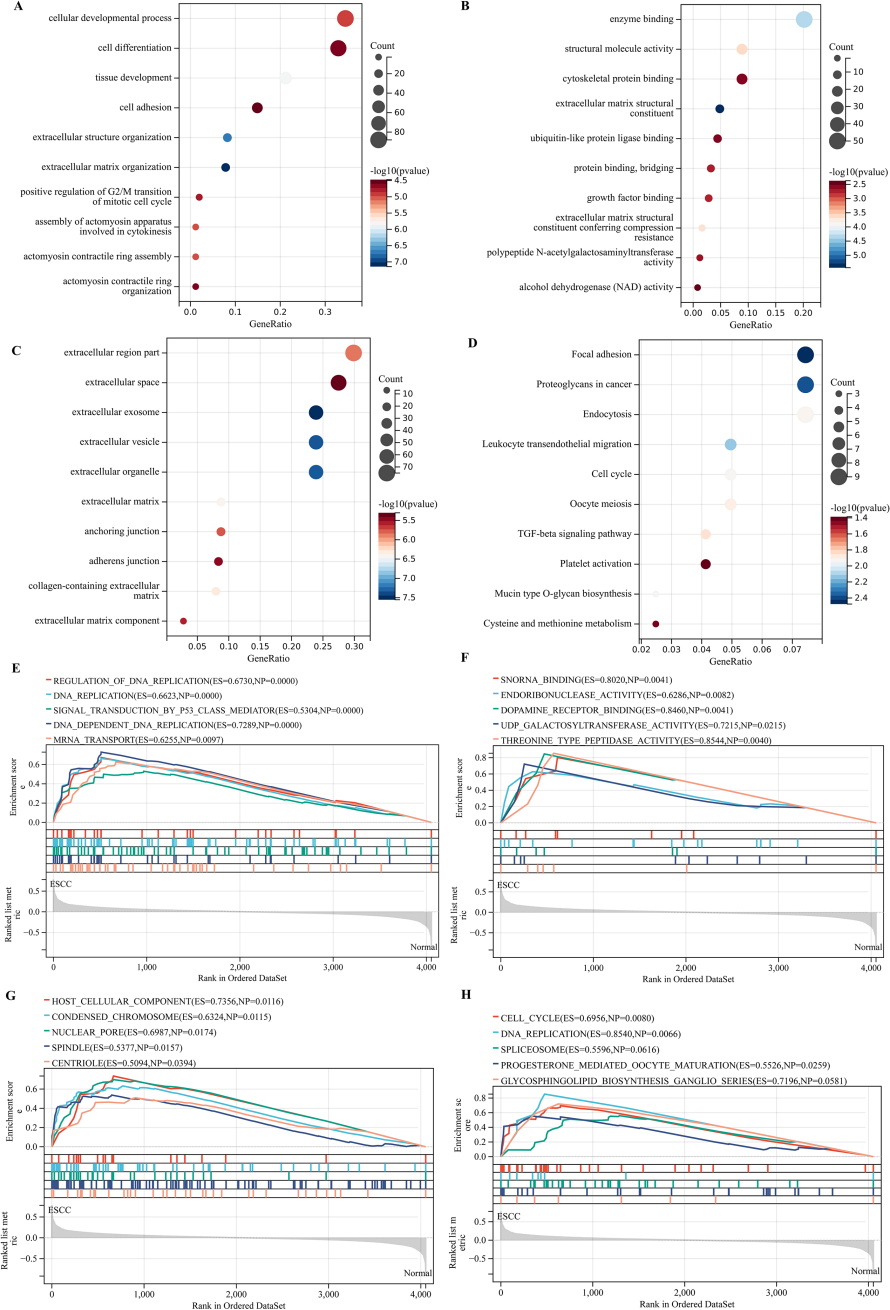
Gene set enrichment analysis (GSEA) for the DEGs and merged dataset. **(A)** Bubble plot for BP on the genes of DEGs. **(B)** Bubble plot for MF on the genes of DEGs. **(C)** Bubble plot for CC on the genes of DEGs. **(D)** Bubble plot for KEGG enrichment analysis on the genes of DEGs. **(E)** Enrichment plot for BP on the merged dataset. **(F)** Enrichment plot for MF on the merged dataset. **(G)** Enrichment plot for CC on the merged dataset. **(H)** Enrichment plot for KEGG on the merged dataset.

We conducted GO and KEGG Enrichment Analysis on the genes of DEGs. In terms of BP, they were mainly enriched in the cell differentiation, cell adhesion, and positive regulation of G2/M transition of mitotic cell cycle (**Figure 3A**). In terms of MF, they were mainly enriched in the cytoskeletal protein binding, ubiquitin-like protein ligase binding and growth factor binding (**Figure 3B**). In terms of CC, they were mainly enriched in the extracellular matrix component, extracellular space, and extracellular matrix component (**Figure 3C**). In terms of KEGG, they were mainly enriched in the Platelet activation, Cysteine and methionine metabolism, and TGF-beta signaling pathway (**Figure 3D**).

Then, the merged dataset was subjected to GSEA (v4.1.0). In terms of BP, the signal transduction by p53 class mediator, DNA-dependent DNA replication, and mRNA transport were all markedly up-regulated in ESCC (**Figure 3E**). In terms of MF, the snoRNA binding, dopamine receptor binding, and threonine-type peptidase activity were all markedly up-regulated in ESCC (**Figure 3F**). In terms of CC, the host cellular component, condensed chromosome, and nuclear pore were all markedly up-regulated in ESCC (**Figure 3G**). In terms of KEGG, the DNA replication, cell cycle, and spliceosome were all markedly up-regulated in ESCC (**Figure 3H**).

### 3.3 The WGCNA analysis revealed a blue module most related to ESCC, comprising 13 genes

**Figure 4:**
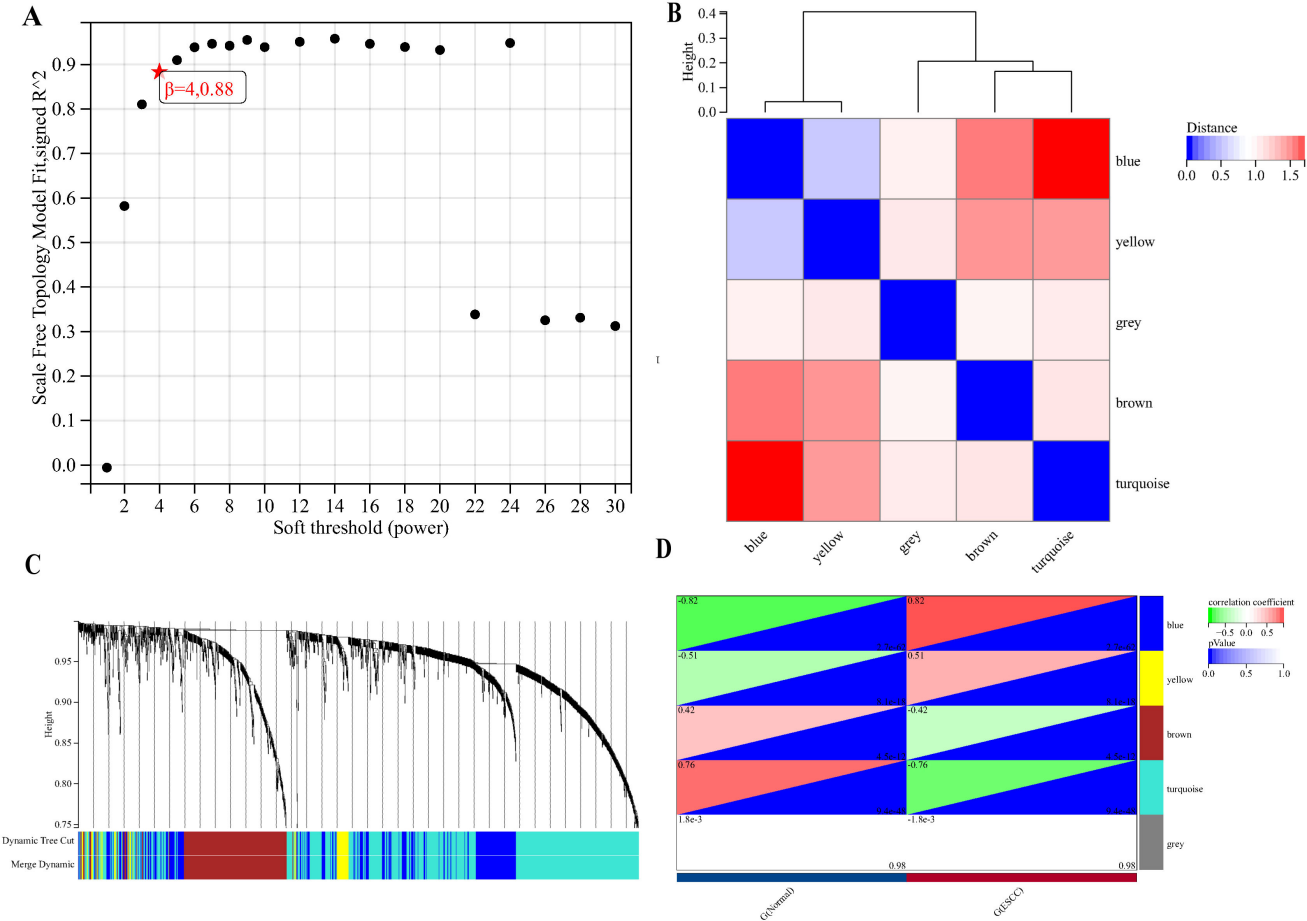
The blue module exhibited the strongest correlation with ESCC. **(A)** The scale-free fit index for soft-thresholding powers. **(B)** Coexpression cluster dendrogram. **(C)** Clustering dendrograms of genes. **(D)** A heatmap showing the correlation between the gene module and ESCC.

The WGCNA package was utilized to establish gene co-expression networks. A scatter plot depicting the fit coefficients for the scale-free topology model was generated (**Figure 4A**), with the red pentagram marking the initial point surpassing the scale-free topology model fit index R²>0.85, associated with a soft threshold β of 4, and an R² value of 0.88. Following the transformation of the correlation matrix into an adjacency matrix using this soft threshold β, a topological overlap matrix and a gene hierarchical clustering dendrogram were assembled. The distances among each module were depicted using a co-expression cluster dendrogram (**Figure 4B**), leading to the identification of 5 gene modules, which are illustrated as clustering dendrograms of genes highlighting the correlations among different models (**Figure 4C**). As depicted in **Figure 4D**, the blue module demonstrated the highest correlation with esophageal squamous cell carcinoma (correlation index: 0.82, P<0.001). This gene model encompasses 13 genes, including *ANP32E*, *ATAD2*, *BID*, *CBX3*, *CCNB1*, *DTL*, *ECT2*, *GMPS*, *KIF14*, *MCM10*, *NDC1*, *NETO2*, and *SORBS2*.

### 3.4 GSEA for the genes in the blue module which were DEGs

**Figure 5:**
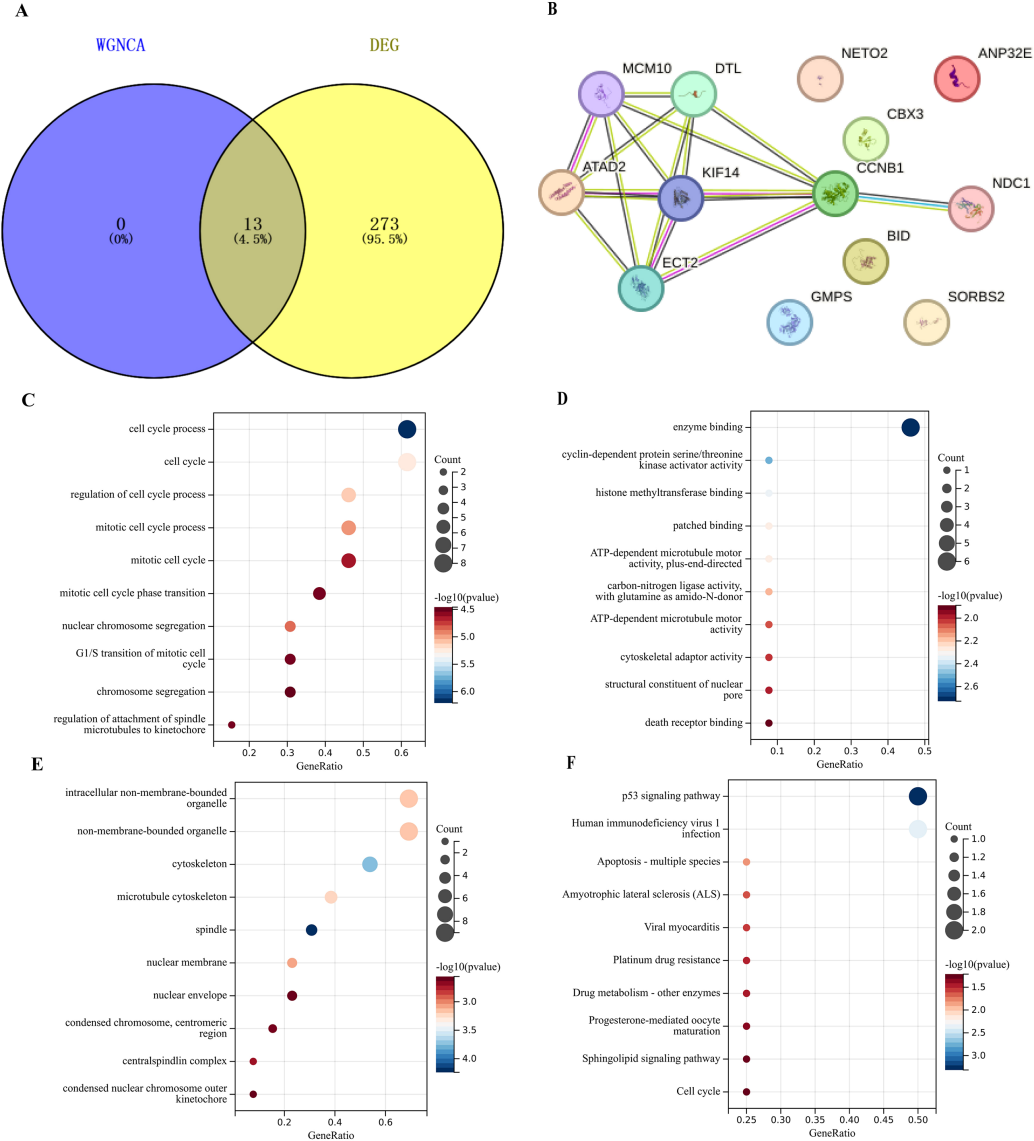
Integrative analysis of the blue module. **(A)** Venn diagram plotting the intersection of WGCNA with DEG. **(B)** The protein–protein interaction (PPI) plot of the genes in the blue module. **(C)** Bubble plot for BP on the genes in the Blue Module. **(D)** Bubble plot for MF on the genes in the Blue Module. **(E)** Bubble plot for CC on the genes in the Blue Module. **(F)** Bubble plot for CC on the genes in the Blue Module.

We intersected the genes in the blue module identified by WGCNA with the related genes in the DEGs and obtained 13 genes (**Figure 5A**). Based on the 13 genes that overlapped between the identified modules and DEGs, we performed PPI network analysis to assess their interactions (**Figure 5B**).

Subsequently, we conducted GSEA on the genes of the blue module identified by WGCNA. In terms of BP, they were mainly enriched in the cell cycle, G1/S transition of mitotic cell cycle, and mitotic cell cycle (**Figure 5E**). In terms of MF, they were mainly enriched in the enzyme binding, cytoskeletal adaptor activity and death receptor bingding (**Figure 5F**). In terms of CC, they were mainly enriched in the cytoskeleton, nuclear membrane, and non-membrane-bounded organelle (**Figure 5G**). In terms of KEGG, they were mainly enriched in the p53 signaling pathway, cell cycle, and sphingolipid signaling pathway (**Figure 5H**).

### 3.5 Construction of ESCC diagnostic model and external validation

**Figure 6:**
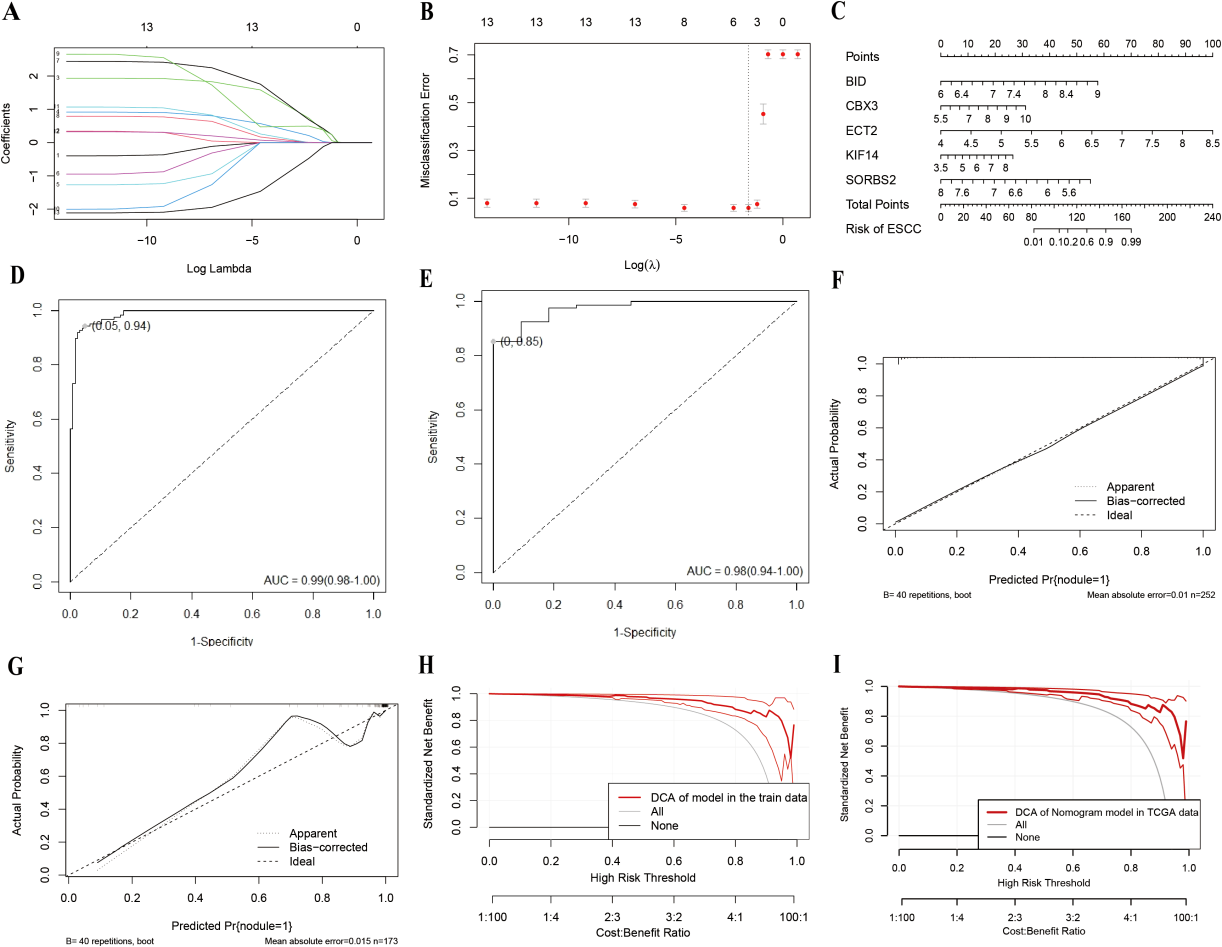
Diagnostic model construction and evaluation. **(A, B)** Construction of the diagnostic model based on the LASSO algorithm. **(C)** Nomogram based on multivariate logistic regression analysis. **(D, E)** ROC curves for training and external validation sets. **(F)** Calibration curve of the training set. **(G)** Calibration curve of the external validation set (TCGA). **(H, I)** Decision Curve Analysis (DCA) plots for training and external validation sets.

The 13 genes in the blue blocks were selected as potential predictors for LASSO regression analysis, and the LASSO coefficient path plot was shown in **Figure 6A**. There are a total of 5 non-zero coefficient genes, including *BID* (coefficient 0.37), *CBX3* (coefficient 0.03), *ECT2* (coefficient 0.42), *KIF14* (coefficient 0.38), and *SORBS2* (coefficient -0.12). The coefficients in the LASSO regression model were visualized in **Figure 6B**. The ESCC diagnostic model was developed using these five genes and analyzed through multivariate logistic regression. In the context of multivariate logistic regression analysis, *SORBS2* emerged as a statistically significant predictor of ESCC (**Table 1**). Our predictive model was visualized as a user-friendly nomogram (**Figure 6C**).

**Table 1:** Multivariate logistic regression analysis of screening diagnostic genes.

Gene	B	Wald	OR (95%CI)	P value
(Intercept)	-24.46	5.83	0 (0~0.002)	0.01
*BID*	2.06	4.87	7.82 (1.44~56.07)	0.03
*CBX3*	0.74	0.46	2.095 (0.28~20.95)	0.50
*ECT2*	2.38	9.28	10.76 (2.45~54.29)	<0.01
*KIF14*	0.57	0.72	1.76 (0.47~6.76)	0.40
*SORBS2*	-2.10	6.34	0.12 (0.02~0.61)	0.01

We used TCGA as an external validation to evaluate the performance of the diagnostic model. The AUC values for the training and test sets were 0.99 (0.98, 1.00) and 0.98 (0.94, 1.00) (**Figures 6D, E**), and the performance of the diagnostic model is presented in **Table 2**, indicating that the model had good accuracy. The calibration curve suggests acceptable model calibration, with good agreement between actual frequencies and predicted probabilities (**Figures 6F, G**). The R²in the training set was 0.724; the bootstrap-internal validation yielded an R² of 0.6663, while the external validation set (TCGA) achieved an R²of 0.702. Ultimately, we generated DCA curves to demonstrate the practical utility of our model in a clinical setting (**Figures 6H, I**). Stratified analysis by ancestry indicated consistent discrimination in Asian (n=25; AUC = 0.95, R² = 0.85, Brier = 0.04) and stable performance in the White subgroup (n=57; AUC = 0.98, R² = 0.84, Brier = 0.04). See **Table 2** for details. Due to the small number of Black or African-American cases (n = 2) and the potentially heterogeneous composition of the Unknown/Mixed group (n = 7), performance metrics for these subgroups are not presented in **Table 2** to avoid unreliable estimates and potential bias.

**Table 2:** Performance of the diagnostic model.

Metric	Training set (N = 252)	TCGA External validation set (N = 91)	TCGA External validation set (N = 91)	
			Asian (N = 25)	White (N = 57)
AUC	0.99 (0.98-1.00)	0.98 (0.94-1.00)	0.95 (0.85–1.00)	0.98 (0.95–1.00)
Accuracy	0.95 (0.95-0.95)	0.87 (0.87-0.87)	0.84 (0.83–0.85)	0.93 (0.93–0.93)
Sensitivity	0.94 (0.90-0.98)	0.85 (0.77-0.93)	0.81 (0.64–0.98)	0.92 (0.85–1.00)
Specificity	0.95 (0.92-0.99)	1.00 (1.00-1.00)	1.00 (1.00–1.00)	1.00 (1.00–1.00)

### 3.6 Experimental verification of signature gene expression in mice ESCC model

**Figure 7:**
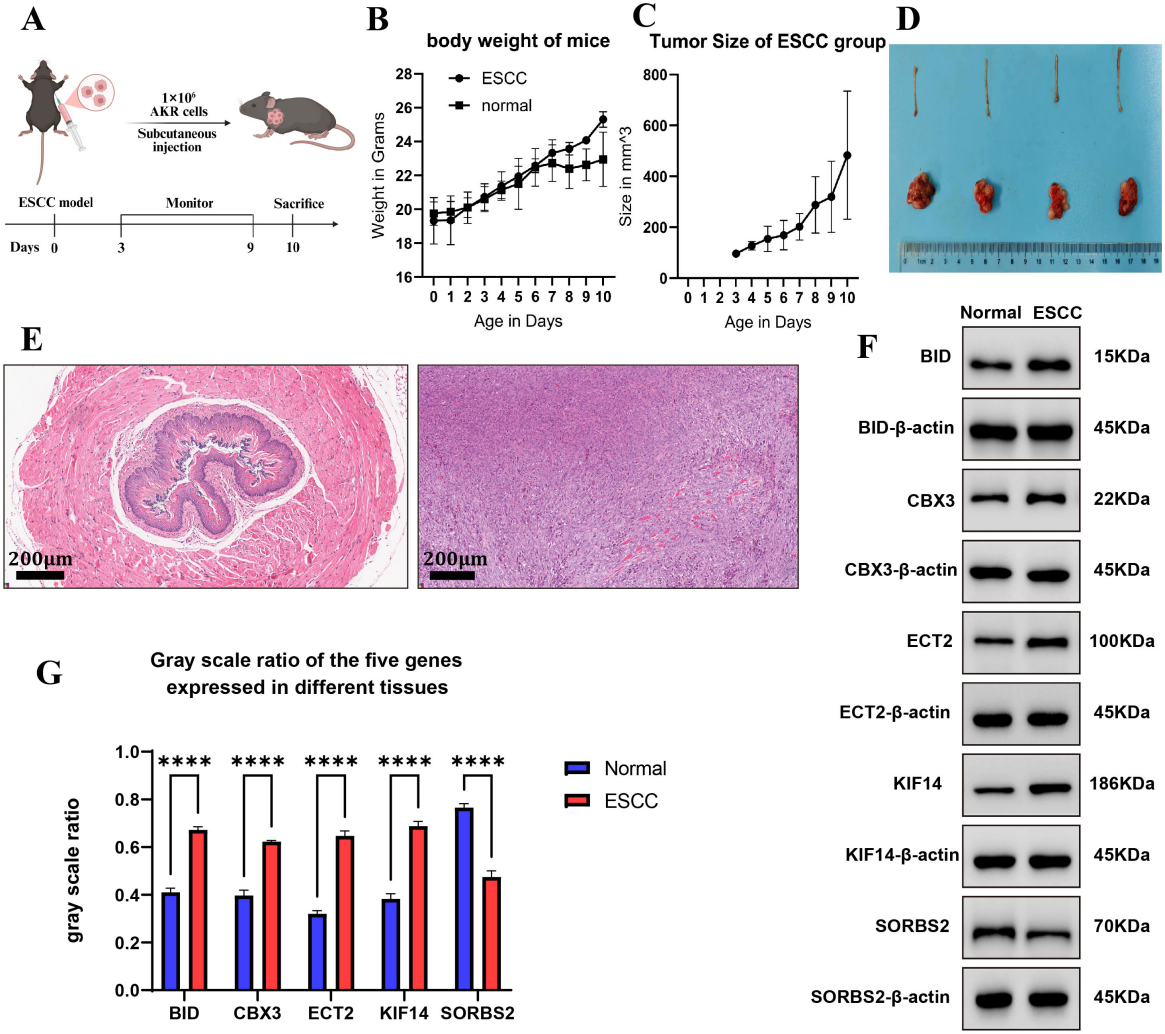
Experimental verification of signature gene expression in mouse ESCC model. **(A)** The modeling process of Mice ESCC Model. **(B)** The body weight of mouse ESCC model and mouse normal model. **(C)** Tumor size of ESCC group, **(D)** Comparison of normal esophageal tissue and ESCC tissue. **(E)** The HE assays of mouse ESCC model and normal model. Magnification: 100x. Scale bar: 200μm **(F)** Western Blot of *BID, CBX3, ECT2, KIF14, SORBS2* expression in ESCC model and normal model. **(G)** The gray scale ratio of the five genes expressed in ESCC model and normal model. ****P < 0.0001.

To confirm the expression differences between ESCC and normal tissues of five genes, we established a Mice ESCC Model (**Figure 7A**). We found that the ESCC model mice didn’t have a significant increase in body weight compared to normal mice (**Figure 7B**). The tumor growth trend of the ESCC model is shown in **Figure 7C**. Comparison of normal esophageal tissue and ESCC tissue is shown in **Figure 7D**. The HE staining revealed disrupted tissue architecture in the ESCC mouse model, characterized by the size and shape of the cells are extremely irregular, and the cell arrangement is disordered compared to normal mice (**Figure 7E**). Subsequently, we performed Western blotting to evaluate the protein levels, which showed a significant increase in *BID*, *CBX3*, *ECT2*, *KIF14* protein expression, and a decrease in *SORBS2* protein expression in ESCC tissues compared to the adjacent normal esophageal tissue (**Figures 7F, G**).

### 3.7 Immune cell infiltration analysis revealed that impaired anti-tumor immunity was correlated with *SORBS2* low expression in the immune microenvironment of ESCC

**Figure 8:**
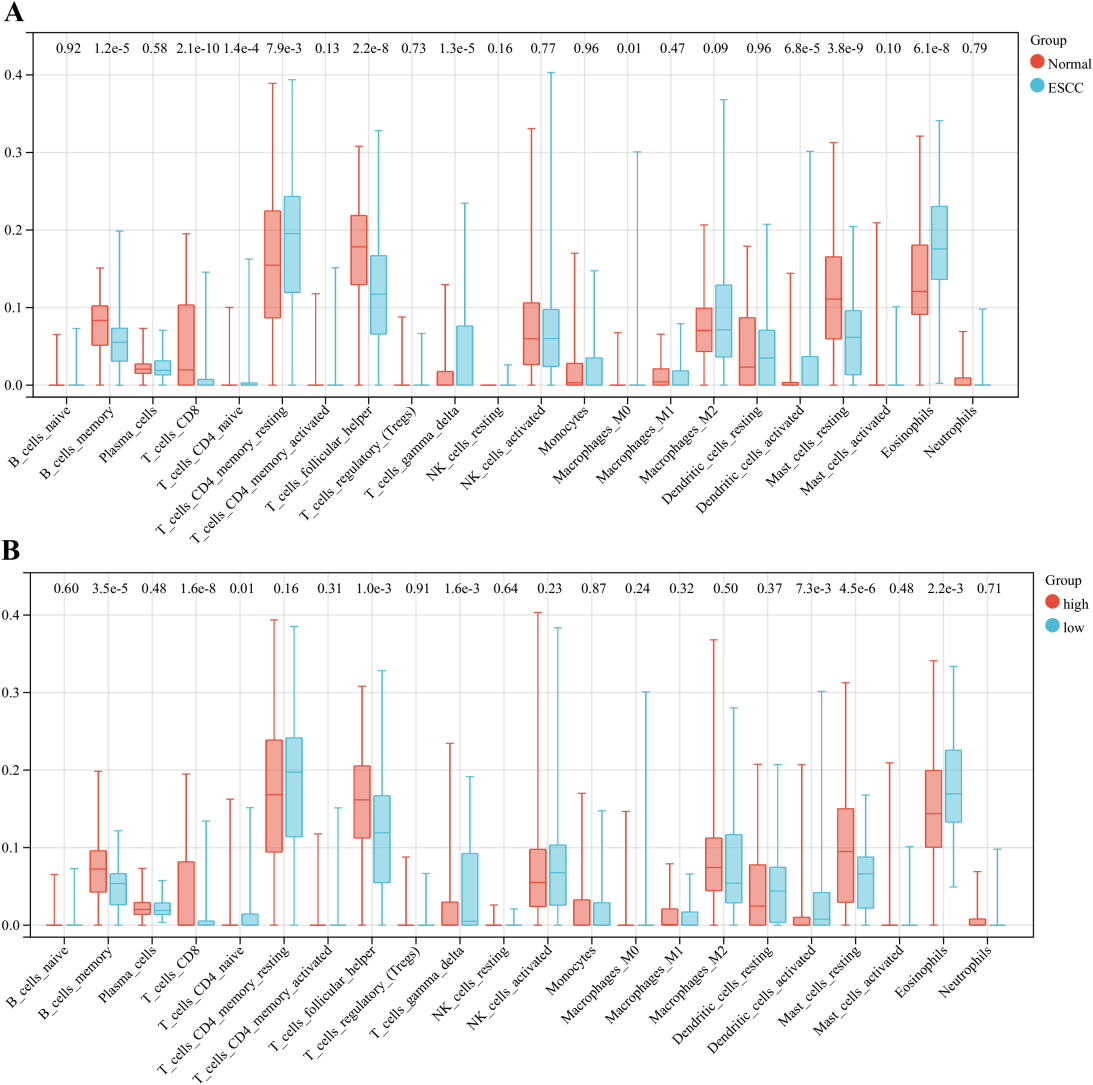
Immune infiltration analysis. **(A)** Expression of different immune cells in ESCC and normal groups. **(B)** Expression of different immune cells in high-expression and low-expression *SORBS2* groups.

We analyzed the IME of ESCC using the CIBERSORT algorithm, which profiles 22 distinct immune cell types. The results showed that out of these 22 cell types, a total of 11 microenvironment cells exhibited significant differences between the normal and ESCC groups (**Figure 8A**). Notably, four lymphocyte populations—memory B cells, CD8+ T cells, follicular helper T cells, and resting mast cells—demonstrated significantly attenuated infiltration within IME relative to normal counterparts. This immunophenotypic landscape indicated profound impairment of anti-tumor immunity. In addition, we analyzed the correlation between *SORBS2* expression and immune cell infiltration in the IME of ESCC, revealing that memory B cells, CD8+ T cells, follicular helper T cells, and resting mast cells attenuated infiltration within the low-expression *SORBS2* group relative to the high-expression *SORBS2* group (**Figure 8B**). Therefore, low expression of *SORBS2* in the IME is associated with the impaired anti-tumor immune function of ESCC.

### 3.8 GSEA revealed the function of *SORBS2* in immune response and cellular signaling pathways

**Figure 9:**
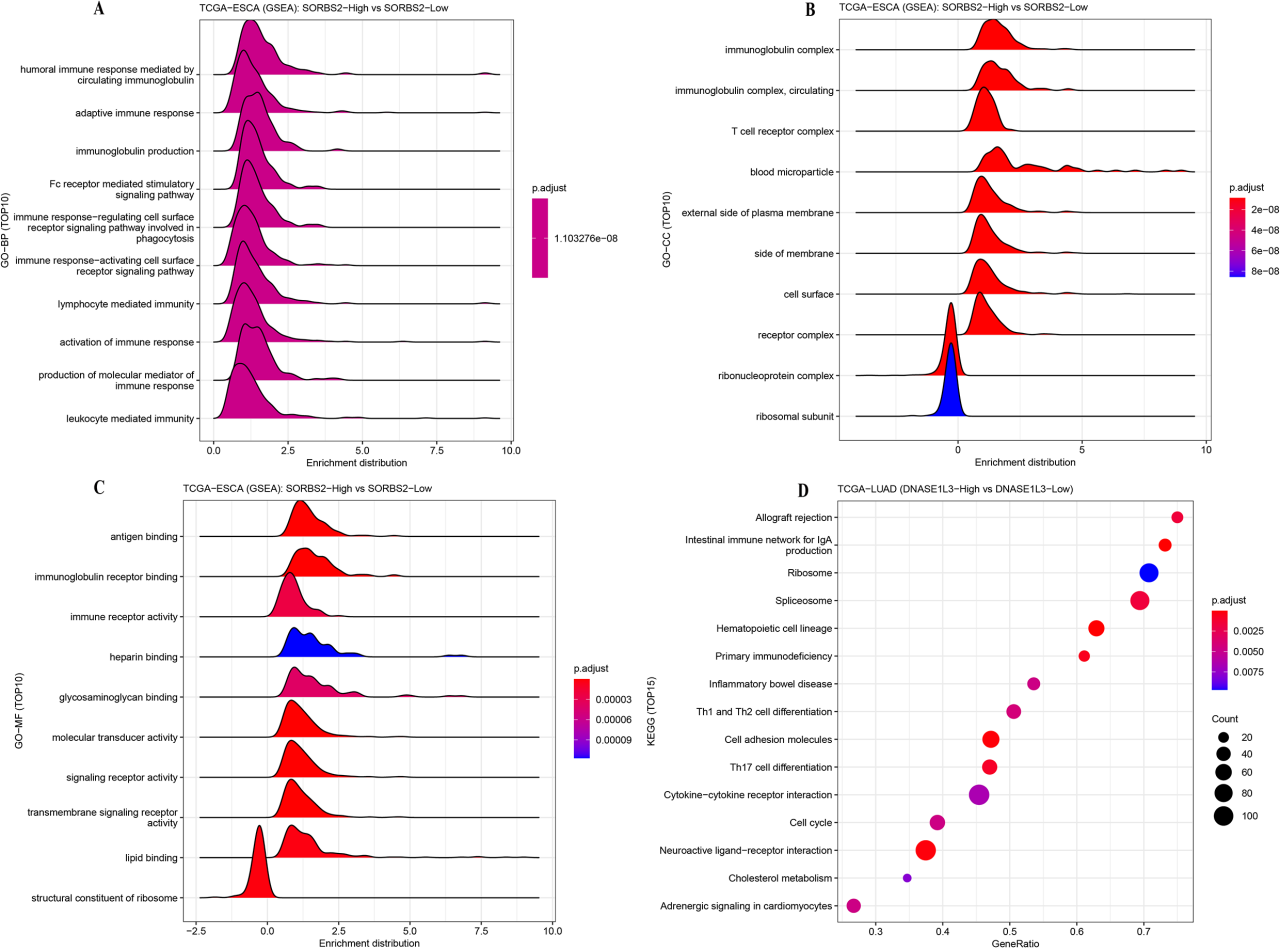
GSEA for *SORBS2*. **(A)** BP. **(B)** CC. **(C)** MF. **(D)** KEGG.

To explore the functional significance of *SORBS2*, we conducted a GSEA. GO enrichment analysis revealing that BP of *SORBS2* was predominantly involved in humoral immune response mediated by circulating immunoglobulin, immune response−regulating cell surface receptor signaling pathway involved in phagocytosis, and immune response−activating cell surface receptor signaling pathway (**Figure 9A**). In terms of CC, *SORBS2* was enriched in the ribonucleoprotein complex, T cell receptor complex, and receptor complex (**Figure 9B**), while at the MF, *SORBS2* was associated with structural constituent of ribosome, immune receptor activity and signaling receptor activity (**Figure 9C**). Utilizing KEGG enrichment analysis, *SORBS2* demonstrated significant relationships with cell adhesion molecules, spliceosome and neuroactive ligand−receptor interaction pathways (**Figure 9D**).

### 3.9 Single-cell analysis revealed predominant *SORBS2* expression in myofibroblast and vascular endothelial cell clusters

**Figure 10:**
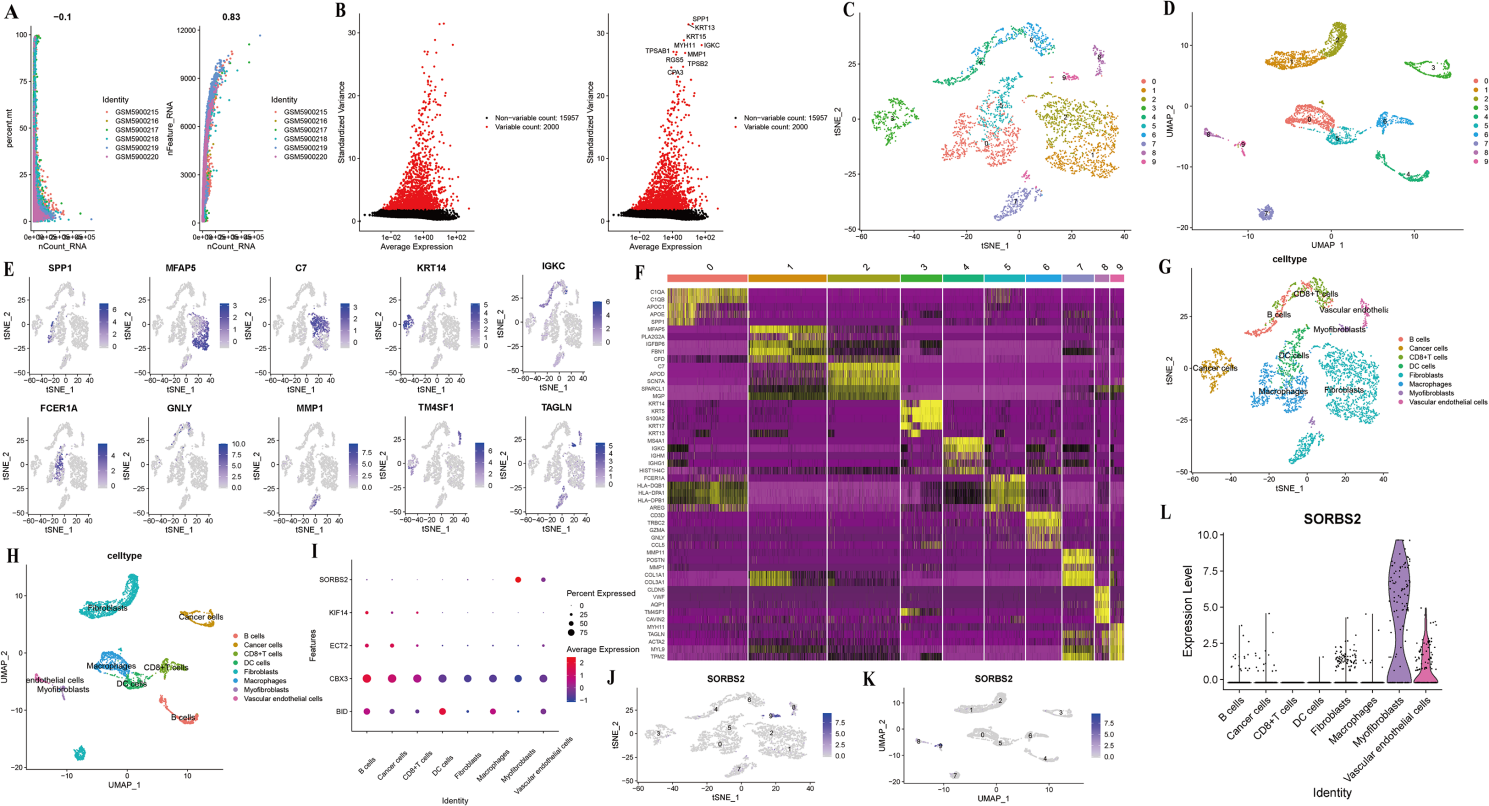
Analysis of single-cell RNA sequencing data of six esophageal samples. **(A)** The correlation for nCount_RNA between percent. Mt and nFeature_RNA. **(B)** Identification of highly variable genes achieved through batch removal post-count. **(C, D)** t-SNE or UMAP plots to identify each cell cluster in esophageal cancer. **(E)** Ten identified marker genes were used to show in different clusters. **(F)** Heatmap to show marker genes of ten cell clusters. **(G, H)** t-SNE or UMAP plots to identify each cell type in esophageal cancer. **(I)** Heatmap to show the expression of five hub genes in eight cell types. **(J-L)** Feature and violin plots to show the distribution of *SORBS2* in various cell types.

Six samples from the GSE196756 database underwent single-cell sequencing analysis, revealing a positive correlation between nCount RNA and nFeature RNA (**Figure 10A**). The variance plot identified 250 genes across all cells, with 2000 highly variable genes marked in red and the top 10 genes labeled (**Figure 10B**). **Figures 10C, D** illustrated that the cells were classified into 10 clusters. **Figure 10E** demonstrated that ten identified marker genes were used to show in different clusters, while the heatmap displays the top 5 marker genes for each cell type (**Figure 10F**). Based on key marker gene expression, these clusters were classified as B cells, cancer cells, CD8 T cells, DC cells, fibroblasts, macrophages, myofibroblasts, and vascular endothelial cells (**Figures 10G, H**). **Figure 10I** illustrates the distribution of five genes across cell types. **Figures 10J-L** indicate predominant *SORBS2* expression in clusters 8 and 9, particularly within myofibroblast and vascular endothelial cell populations.

### 3.10 Inferences of cell–cell communication by cellchat revealed the signaling pathways involving CDH5 and PECAM1 in vascular endothelial cells and myofibroblasts

**Figure 11:**
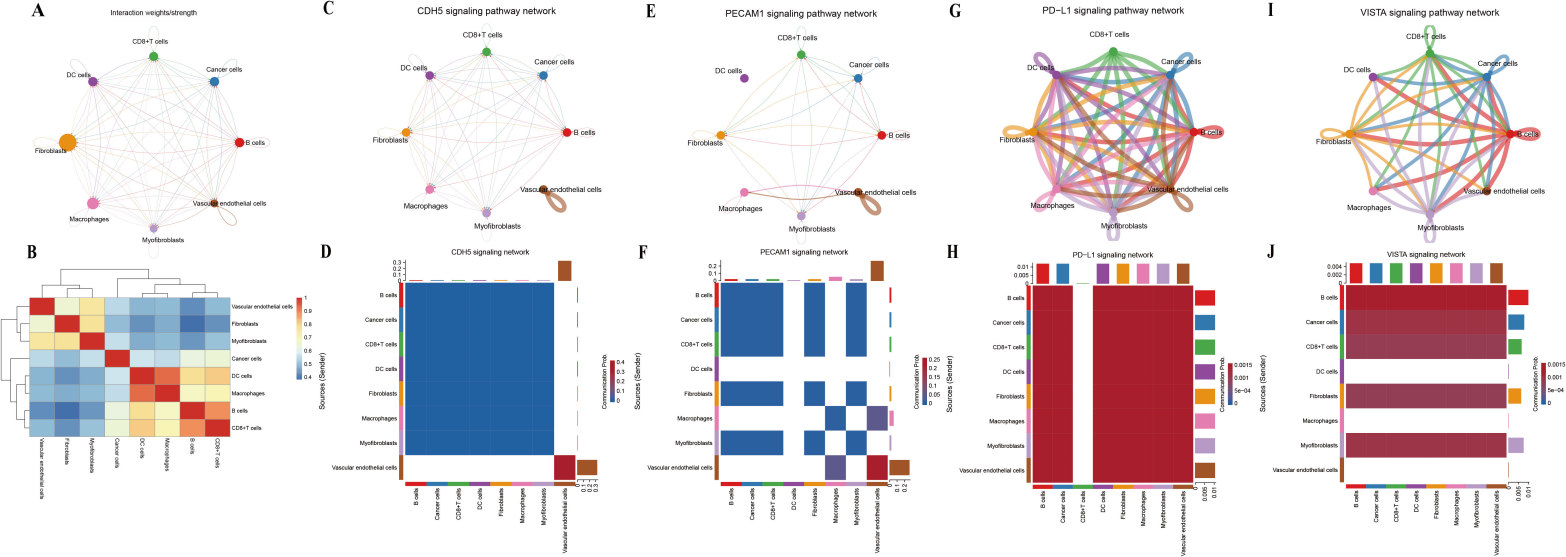
Inferences of cell–cell communication by cellchat. **(A)** CellChat network depicting interaction strengths. **(B)** Heatmap showing the correlation between different cell types. **(C, D)** The communication between different cell types is mediated by CDH5 signaling pathways. **(E, F)** The communication between different cell types is mediated by PECAM1 signaling pathways. **(G, H)** The communication between different cell types is mediated by PD-L1 signaling pathways. **(I, J)** The communication between different cell types is mediated by VISTA signaling pathways.

The circular plots of the cellular interaction strength between each cell type were shown in **Figure 11A**. There was a high correlation between vascular endothelial cells and myofibroblasts (**Figure 11B**). We further investigated the signaling network of vascular endothelial cells and myofibroblast-related molecules, including CDH5 and PECAM1. In the CDH5 signaling network, vascular endothelial cells and myofibroblasts are the main signal transmitters and have a close relationship with cancer cells (**Figures 11C, D**). The PECAM1 signaling network also shows that vascular endothelium and myofibroblasts have a close relationship with cancer cells (**Figures 11E, F**). In addition, we investigated immune-related molecular signaling pathways, including PD-L1 and VISTA. The results showed that Myofibroblasts have a relatively close relationship with cancer cells in the PD-L1 signaling network and mainly interact significantly with cancer cells and CD8+ T cells in the VISTA signaling network (**Figures 11G-J**).

### 3.11 Human ESCC tissue immunofluorescence review *SORBS2* a lower expression in ESCC tumor tissue

**Figure 12:**
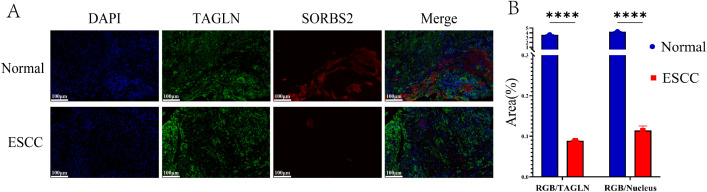
Human ESCC Tissue Immunofluorescence. **(A)** The expression of DAPI (blue, nucleus), *TAGLN* (green), and *SORBS2* (red) in normal and ESCC cells. The merged image (Merge) displays the co-localization of *TAGLN, SORBS2*, and DAPI. Magnification: 400x. Scale bar: 100μm. **(B)** Bar graphs show the relative area percentage of *TAGLN* and *SORBS2* in cells, as well as the co-localization. RGB: RED+GREEN+BLUE. RGB/TAGLN: the proportion of cells expressing *TAGLN* among all cells expressing all three colors; RGB/Nucleus: the proportion of nucleated cells among all cells expressing all three colors. ****P < 0.0001.

As it is shown in **Figure 12A**, DAPI (blue) marked all nucleated cells, *TAGLN* (green) identified myofibroblasts, and *SORBS2* (red) highlighted cells expressing this tumor-suppressive protein. The expression of *SORBS2* is higher in adjacent normal tissues compared to ESCC tissues. Moreover, in adjacent normal tissues, *SORBS2* shows stronger co-localization with myofibroblasts than in ESCC (**Figure 12B**).

## 4 Discussion

In this study, we integrated DEG and WGCNA analyses to identify 13 key differentially expressed genes most strongly associated with ESCC, including *ANP32E*, *ATAD2*, *BID*, *CBX3*, *CCNB1*, *DTL*, *ECT2*, *GMPS*, *KIF14*, *MCM10*, *NDC1*, *NETO2*, and *SORBS2*. We screened out five genes with non-zero gene coefficients, including *BID*, *CBX3*, *ECT2*, *KIF4*, and *SORBS2*, through the LASSO algorithm. *BID* acted as a crucial link between death receptor-initiated signals and the core mitochondrial pathway of apoptosis, adept at orchestrating a suite of cellular functions, including apoptosis, survival, and proliferation of tumor cells (20). *BID* was now on the radar for its apoptotic superpowers over gastric, liver, and esophageal cancer cells (21, 22). In the case of esophageal cancer cells, pro-apoptotic *BCL-2* family proteins, including *BID*, translocated to the mitochondrial outer membrane, sparking the release of pro-apoptotic factors and precipitating cellular suicide (23). Chromebox protein homolog 3 (*CBX3*), a member of the heterochromatin-associated protein 1 (*HP1*) family, has been detected as overexpressed in EC (24). Overexpression of *CBX3* enhances cell proliferation and migration while suppressing apoptosis in ESCC via the *JAK2/STAT3* signaling pathway (25). Conversely, *CBX3* inhibition activated the P53/P21 pathway, impairing the self-renewal capacity of ESCC stem cells (26). The expression of *ECT2* (Epithelial Cell Transforming Sequence 2) was upregulated in ESCC, and its elevated expression has been correlated with enhanced proliferation, colony formation, migration, and invasion capabilities of ESCC cells, as well as a reduction in apoptotic rates (27–29). Additionally, a significant correlation existed between *ECT2* expression levels and patient prognosis. Higher expression levels were associated with worse prognoses (30). *KIF14*(Kinesin Family Member 14), as a microtubule motor protein, with its expressed at levels that were closely associated with the phenotype of cancer cells (31, 32). *KIF14* was one of the most significant central genes implicated in the development of ESCC through hypermethylation (33). A decrease in its expression level could not only inhibit the proliferation, invasion, migration, and angiogenesis of ESCC cells (34), but also might enhance the sensitivity of tumors to chemotherapy drugs (35). *SORBS2* was an RNA-binding protein that could inhibit the migration and invasion of hepatocellular carcinoma and colorectal cancer cells (36–38), indicating that *SORBS2* might have a protective effect on malignant tumors of the digestive system. This finding was consistent with our research conclusion that *SORBS2* was a protective factor for ESCC.

Next, we constructed a predictive model using multivariate logistic regression. This model demonstrated significant efficacy in both the training and external validation sets, with an area under the curve (AUC) value exceeding 0.9. This indicated that our model possessed outstanding predictive capability. The R² values were 0.724 in the training set, 0.6663 in bootstrap-internal validation, and 0.702 in the external TCGA cohort. This agreement indicated minimal overfitting and strong generalizability of the model. We then created a diagnostic nomogram to illustrate the diagnostic model. The calibration curve showed good consistency between the actual frequency and the predicted probability, suggesting that the diagnostic model was highly accurate. Additionally, *SORBS2* was the only protective factor for ESCC and a statistically significant predictive factor, with an odds ratio (OR) of 0.12.

In the ESCC mouse model, our study revealed that *BID*, *CBX3*, *ECT2*, and *KIF14* expression levels were significantly higher in ESCC tissues than in adjacent normal esophageal tissues, while *SORBS2* expression was significantly downregulated.

Finally, we performed a single-cell RNA analysis. The results showed that *SORBS2* was predominantly expressed in myofibroblast and vascular endothelial cell populations. CDH5 and PECAM1 were both related to vascular endothelial cells and myofibroblasts. Their signaling networks have shown that vascular endothelial cells and myofibroblasts were closely associated with cancer cells. A recent study has shown that *SORBS2* reduced the degradation of the extracellular matrix, angiogenesis, and the invasion and metastasis of tumors in ESCC by upregulating TIMP and inhibiting MMP activity (39). Additionally, in lung adenocarcinoma, MMP9 was upregulated while *CDH5* and *PECAM1* were downregulated, resulting in vascular abnormalities (40). Therefore, low expression of SORBS2 was associated with altered activity of the CDH5 and PECAM1 signaling pathways, suggesting its potential role in angiogenesis and stromal remodeling in the tumor microenvironment, which might contribute to attenuated tumor invasion and metastasis. In addition, myofibroblasts had a strong interaction relationship with cancer cells and CD8^+^ T cells in the PD-L1 signaling network and the VISTA signaling network. As we all know, PD-L1 and VISTA were immune checkpoint molecules that could interact with cell surface receptors, regulate immune responses, and play an important role in tumor immune escape (41, 42). Studies have shown that myofibroblasts could overexpress PD-L1 and VISTA in cancer tissue (43–45). The results of immune infiltration analysis and GSEA indicated that high expression of SORBS2 was correlated with a less immunosuppressive tumor microenvironment and was enriched in immune response–activating cell surface receptor signaling pathways, suggesting its potential involvement in anti-tumor immune regulation.

However, our study still had some limitations. First, our data came from publicly available datasets, which might have sample selection and ethnographic bias. Across the two largest ancestry subgroups (Asian and White), we successfully validated the model’s predictive efficacy in the Asian and White populations within the TCGA database, indicating stable model predictive performance; we would conduct large, multi-center cohorts to confirm generalizability across all ancestries. Secondly, we have not conducted further mechanistic investigations to elucidate how these five genes drive the initiation and progression of ESCC. To further verify the role of SORBS2 in promoting antitumor immunity, we will conduct *in vitro* and *in vivo* experiments. Specifically, we will knock out or overexpress the SORBS2 gene to observe the changes in cellular behavior. In mouse tumor models, we will knock out or overexpress SORBS2 to investigate the changes in tumor growth and immune cell infiltration. In the future, we will launch a prospective cohort study to externally validate the predictive performance of the model in a large, independent population. For clinical translation, the five-gene signature would be detected in endoscopic biopsy specimens via a practical RT-qPCR assay.

## 5 Conclusion

Our study developed a highly accurate ESCC diagnostic model in the Asian population. Additionally, our findings suggested that high expression of SORBS2 was associated with a tumor microenvironment that favored anti-tumor immunity, potentially involving immune response-related cell-surface receptor signaling pathways.

## Data Availability

The original contributions presented in the study are included in the article/supplementary material. Further inquiries can be directed to the corresponding author/s.

## References

[1] Sung H Ferlay J Siegel RL Laversanne M Soerjomataram I Jemal A . Global cancer statistics 2020: globocan estimates of incidence and mortality worldwide for 36 cancers in 185 countries. CA: A Cancer J Clin. (2021) 71:209–49. doi: 10.3322/caac.21660, PMID: 33538338

[2] Cao W Chen HD Yu YW Li N Chen WQ . Changing profiles of cancer burden worldwide and in China: A secondary analysis of the global cancer statistics 2020. Chin Med J. (2021) 134:783–91. doi: 10.1097/cm9.0000000000001474, PMID: 33734139 PMC8104205

[3] Sun G Yang Y Liu J Gao Z Xu T Chai J . Cancer stem cells in esophageal squamous cell carcinoma. Pathol Res Pract. (2022) 237:154043. doi: 10.1016/j.prp.2022.154043, PMID: 35926434

[4] Zeng H Chen W Zheng R Zhang S Ji JS Zou X . Changing cancer survival in China during 2003-15: A pooled analysis of 17 population-based cancer registries. Lancet Global Health. (2018) 6:e555–e67. doi: 10.1016/s2214-109x(18)30127-x, PMID: 29653628

[5] Soares-Lima SC Gonzaga IM Camuzi D Nicolau-Neto P Vieira da Silva R Guaraldi S . Il6 and bcl3 expression are potential biomarkers in esophageal squamous cell carcinoma. Front Oncol. (2021) 11:722417. doi: 10.3389/fonc.2021.722417, PMID: 34422669 PMC8371528

[6] Zhang J Zhu Z Liu Y Jin X Xu Z Yu Q . Diagnostic value of multiple tumor markers for patients with esophageal carcinoma. PloS One. (2015) 10:e0116951. doi: 10.1371/journal.pone.0116951, PMID: 25693076 PMC4333286

[7] Mroczko B Kozłowski M Groblewska M Łukaszewicz M Nikliński J Jelski W . The diagnostic value of the measurement of matrix metalloproteinase 9 (Mmp-9), squamous cell cancer antigen (Scc) and carcinoembryonic antigen (Cea) in the sera of esophageal cancer patients. Clin Chim Acta Int J Clin Chem. (2008) 389:61–6. doi: 10.1016/j.cca.2007.11.023, PMID: 18155162

[8] Wang C Guan S Liu F Chen X Han L Wang D . Prognostic and diagnostic potential of mir-146a in oesophageal squamous cell carcinoma. Br J Cancer. (2016) 114:290–7. doi: 10.1038/bjc.2015.463, PMID: 26794279 PMC4742585

[9] Takeshita N Hoshino I Mori M Akutsu Y Hanari N Yoneyama Y . Serum microrna expression profile: mir-1246 as a novel diagnostic and prognostic biomarker for oesophageal squamous cell carcinoma. Br J Cancer. (2013) 108:644–52. doi: 10.1038/bjc.2013.8, PMID: 23361059 PMC3593570

[10] Spratt DE Chan T Waldron L Speers C Feng FY Ogunwobi OO . Racial/ethnic disparities in genomic sequencing. JAMA Oncol. (2016) 2:1070–4. doi: 10.1001/jamaoncol.2016.1854, PMID: 27366979 PMC5123755

[11] Lin Y Totsuka Y He Y Kikuchi S Qiao Y Ueda J . Epidemiology of esophageal cancer in Japan and China. J Epidemiol. (2013) 23:233–42. doi: 10.2188/jea.je20120162, PMID: 23629646 PMC3709543

[12] Su H Hu N Yang HH Wang C Takikita M Wang QH . Global gene expression profiling and validation in esophageal squamous cell carcinoma and its association with clinical phenotypes. Clin Cancer Res. (2011) 17:2955–66. doi: 10.1158/1078-0432.ccr-10-2724, PMID: 21385931 PMC3086948

[13] Lee JJ Natsuizaka M Ohashi S Wong GS Takaoka M Michaylira CZ . Hypoxia activates the cyclooxygenase-2-prostaglandin E synthase axis. Carcinogenesis. (2010) 31:427–34. doi: 10.1093/carcin/bgp326, PMID: 20042640 PMC2832548

[14] Hu N Clifford RJ Yang HH Wang C Goldstein AM Ding T . Genome wide analysis of DNA copy number neutral loss of heterozygosity (Cnnloh) and its relation to gene expression in esophageal squamous cell carcinoma. BMC Genomics. (2010) 11:576. doi: 10.1186/1471-2164-11-576, PMID: 20955586 PMC3091724

[15] Naini BV Souza RF Odze RD . Barrett’s esophagus: A comprehensive and contemporary review for pathologists. Am J Surg Pathol. (2016) 40:e45–66. doi: 10.1097/pas.0000000000000598, PMID: 26813745 PMC4833583

[16] Bakr O Zhao W Corley D . Gastroesophageal reflux frequency, severity, age of onset, family history and acid suppressive therapy predict barrett esophagus in a large population. J Clin Gastroenterol. (2018) 52:873–9. doi: 10.1097/mcg.0000000000000983, PMID: 29356784 PMC6053338

[17] Taminau J Meganck S Lazar C Steenhoff D Coletta A Molter C . Unlocking the potential of publicly available microarray data using insilicodb and insilicomerging R/bioconductor packages. BMC Bioinf. (2012) 13:335. doi: 10.1186/1471-2105-13-335, PMID: 23259851 PMC3568420

[18] Rubenstein JH McConnell D Waljee AK Metko V Nofz K Khodadost M . Validation and comparison of tools for selecting individuals to screen for barrett’s esophagus and early neoplasia. Gastroenterology. (2020) 158:2082–92. doi: 10.1053/j.gastro.2020.02.037, PMID: 32119928 PMC7282990

[19] Mari L Milano F Parikh K Straub D Everts V Hoeben KK . A psmad/cdx2 complex is essential for the intestinalization of epithelial metaplasia. Cell Rep. (2014) 7:1197–210. doi: 10.1016/j.celrep.2014.03.074, PMID: 24794431

[20] Song G Chen GG Hu T Lai PB . Bid stands at the crossroad of stress-response pathways. Curr Cancer Drug Targets. (2010) 10:584–92. doi: 10.2174/156800910791859515, PMID: 20482490

[21] Hu C Song G Zhang B Liu Z Chen R Zhang H . Intestinal metabolite compound K of panaxoside inhibits the growth of gastric carcinoma by augmenting apoptosis via bid-mediated mitochondrial pathway. J Cell Mol Med. (2012) 16:96–106. doi: 10.1111/j.1582-4934.2011.01278.x, PMID: 21323864 PMC3823096

[22] Barathan M Mariappan V Shankar EM Abdullah BJ Goh KL Vadivelu J . Hypericin-photodynamic therapy leads to interleukin-6 secretion by hepg2 cells and their apoptosis via recruitment of bh3 interacting-domain death agonist and caspases. Cell Death Dis. (2013) 4:e697. doi: 10.1038/cddis.2013.219, PMID: 23807226 PMC3702308

[23] Wang Q Du H Geng G Zhou H Xu M Cao H . Matrine inhibits proliferation and induces apoptosis via bid-mediated mitochondrial pathway in esophageal cancer cells. Mol Biol Rep. (2014) 41:3009–20. doi: 10.1007/s11033-014-3160-3, PMID: 24510386

[24] Hou J Yang Y Gao H Ouyang T Liu Q Ding R . Systematic investigation of the clinical significance and prognostic value of the cbxs in esophageal cancer. Medicine. (2022) 101:e30888. doi: 10.1097/md.0000000000030888, PMID: 36221371 PMC9542684

[25] Meng L Wang F Sun S Zheng Y Ding Z Sun Y . Microrna-30b targets cbx3 and regulates cell proliferation, apoptosis, and migration in esophageal squamous cell carcinoma via the jak2/stat3 signaling pathway. Int J Clin Exp Pathol. (2017) 10:11828–37., PMID: 31966547 PMC6966038

[26] He Z Chen J Chen X Wang H Tang L Han C . Microrna-377 acts as a suppressor in esophageal squamous cell carcinoma through cbx3-dependent P53/P21 pathway. J Cell Physiol. (2021) 236:107–20. doi: 10.1002/jcp.29631, PMID: 33459391

[27] Li Y Xu F Chen F Chen Y Ge D Zhang S . Transcriptomics based multi-dimensional characterization and drug screen in esophageal squamous cell carcinoma. EBioMedicine. (2021) 70:103510. doi: 10.1016/j.ebiom.2021.103510, PMID: 34365093 PMC8353400

[28] Sun BY Wei QQ Liu CX Zhang L Luo G Li T . Ect2 promotes proliferation and metastasis of esophageal squamous cell carcinoma via the rhoa-erk signaling pathway. Eur Rev Med Pharmacol Sci. (2020) 24:7991–8000. doi: 10.26355/eurrev_202008_22482, PMID: 32767325

[29] Qixing M Gaochao D Wenjie X Anpeng W Bing C Weidong M . Microarray analyses reveal genes related to progression and prognosis of esophageal squamous cell carcinoma. Oncotarget. (2017) 8:78838–50. doi: 10.18632/oncotarget.20232, PMID: 29108269 PMC5668002

[30] Hirata D Yamabuki T Miki D Ito T Tsuchiya E Fujita M . Involvement of epithelial cell transforming sequence-2 oncoantigen in lung and esophageal cancer progression. Clin Cancer Res. (2009) 15:256–66. doi: 10.1158/1078-0432.ccr-08-1672, PMID: 19118053

[31] Zhu C Zhao J Bibikova M Leverson JD Bossy-Wetzel E Fan JB . Functional analysis of human microtubule-based motor proteins, the kinesins and dyneins, in mitosis/cytokinesis using rna interference. Mol Biol Cell. (2005) 16:3187–99. doi: 10.1091/mbc.e05-02-0167, PMID: 15843429 PMC1165403

[32] Thériault BL Pajovic S Bernardini MQ Shaw PA Gallie BL . Kinesin family member 14: an independent prognostic marker and potential therapeutic target for ovarian cancer. Int J Cancer. (2012) 130:1844–54. doi: 10.1002/ijc.26189, PMID: 21618518

[33] Han BA Yang XP Hosseini DK Zhang P Zhang Y Yu JT . Identification of candidate aberrantly methylated and differentially expressed genes in esophageal squamous cell carcinoma. Sci Rep. (2020) 10:9735. doi: 10.1038/s41598-020-66847-4, PMID: 32546690 PMC7297810

[34] Zhao Q Chen S Chen L . Letm1 (Leucine zipper-ef-hand-containing transmembrane protein 1) silence reduces the proliferation, invasion, migration and angiogenesis in esophageal squamous cell carcinoma via kif14 (Kinesin family member 14). Bioengineered. (2021) 12:7656–65. doi: 10.1080/21655979.2021.1982275, PMID: 34605738 PMC8806762

[35] Corson TW Huang A Tsao MS Gallie BL . Kif14 is a candidate oncogene in the 1q minimal region of genomic gain in multiple cancers. Oncogene. (2005) 24:4741–53. doi: 10.1038/sj.onc.1208641, PMID: 15897902

[36] Yan B Peng Z Xing C . Sorbs2, mediated by mef2d, suppresses the metastasis of human hepatocellular carcinoma by inhibitiing the C-abl-erk signaling pathway. Am J Cancer Res. (2019) 9:2706–18., PMID: 31911856 PMC6943356

[37] Zhang T Chen S Peng Y Wang C Cheng X Zhao R . Nova1-mediated sorbs2 isoform promotes colorectal cancer migration by activating the notch pathway. Front Cell Dev Biol. (2021) 9:673873. doi: 10.3389/fcell.2021.673873, PMID: 34692669 PMC8531477

[38] Han L Huang C Zhang S . The rna-binding protein sorbs2 suppresses hepatocellular carcinoma tumourigenesis and metastasis by stabilizing rora mrna. Liv Int. (2019) 39:2190–203. doi: 10.1111/liv.14202, PMID: 31365778

[39] Zhang W Zhang P Wang X Lin Y Xu H Mao R . Sorbs2-mediated inhibition of Malignant behaviors in esophageal squamous cell carcinoma through timp3. Int Immunopharmacol. (2024) 142:113096. doi: 10.1016/j.intimp.2024.113096, PMID: 39288625

[40] Isıyel M Ceylan H Demir Y . Bioinformatics-based discovery of therapeutic targets in cadmium-induced lung adenocarcinoma: the role of oxyresveratrol. Biol Trace Elem Res. (2026) 204:1068–83. doi: 10.1007/s12011-025-04730-x, PMID: 40610695 PMC12847123

[41] Zuazo M Gato-Cañas M Llorente N Ibañez-Vea M Arasanz H Kochan G . Molecular mechanisms of programmed cell death-1 dependent T cell suppression: relevance for immunotherapy. Ann Trans Med. (2017) 5:385. doi: 10.21037/atm.2017.06.11, PMID: 29114543 PMC5653513

[42] Wang SJ Dougan SK Dougan M . Immune mechanisms of toxicity from checkpoint inhibitors. Trends Cancer. (2023) 9:543–53. doi: 10.1016/j.trecan.2023.04.002, PMID: 37117135 PMC10330206

[43] Hosseinkhani N Derakhshani A Shadbad MA Argentiero A Racanelli V Kazemi T . The role of V-domain ig suppressor of T cell activation (Vista) in cancer therapy: lessons learned and the road ahead. Front Immunol. (2021) 12:676181. doi: 10.3389/fimmu.2021.676181, PMID: 34093577 PMC8172140

[44] Nurmik M Ullmann P Rodriguez F Haan S Letellier E . In search of definitions: cancer-associated fibroblasts and their markers. Int J Cancer. (2020) 146:895–905. doi: 10.1002/ijc.32193, PMID: 30734283 PMC6972582

[45] Kobayashi H Enomoto A Woods SL Burt AD Takahashi M Worthley DL . Cancer-associated fibroblasts in gastrointestinal cancer. Nat Rev Gastroenterol Hepatol. (2019) 16:282–95. doi: 10.1038/s41575-019-0115-0, PMID: 30778141

